# RETRACTED: Ai et al. The Effects of Environmental Regulations on Medical Expenses: Evidence from China. *Int. J. Environ. Res. Public Health* **2022**, *19*, 7567

**DOI:** 10.3390/ijerph21030275

**Published:** 2024-02-28

**Authors:** Hongshan Ai, Xiaoqing Tan, Zhen Xia

**Affiliations:** 1 School of Economics and Trade, Hunan University, Changsha 410079, China; aihongshan@hnu.edu.cn; 2 School of Biomedical Sciences, Hunan University, Changsha 410082, China

The authors and journal retract the following article: “The Effects of Environmental Regulations on Medical Expenses: Evidence from China” [1]. 

Following publication, the authors contacted the Editorial Office regarding concerns relating to the publication of data without appropriate permission and personal privacy of medical expenditures.

Adhering to our complaints procedure, an investigation was conducted by the Editorial Office and Editorial Board which confirmed the failure of the authors to gain appropriate permission from the Hunan Provincial Medical Insurance Bureau to publish the data contained within this article [1]. As a result, the Editorial Office and Editorial Board have decided to retract this article [1] as per MDPI’s retraction policy (https://www.mdpi.com/ethics#_bookmark30). 

This retraction was approved by the Editor-in-Chief of the *International Journal of Environmental Research and Public Health*. 

The authors agreed to this retraction. 
